# Selective Regulation of B-Raf Dependent K-Ras/Mitogen-Activated Protein by Natural Occurring Multi-kinase Inhibitors in Cancer Cells

**DOI:** 10.3389/fonc.2019.01220

**Published:** 2019-11-12

**Authors:** Ahmed I. Abd El Maksoud, Rehab F. Taher, Ahmed H. Gaara, Eman Abdelrazik, Omar S. Keshk, Khaled A. Elawdan, Salwa E. Morsy, Ahmed Salah, Hany Khalil

**Affiliations:** ^1^Industrial Biotechnology Department, Genetic Engineering and Biotechnology Research Institute, University of Sadat City, Sadat City, Egypt; ^2^Natural Compounds Chemistry Department, National Research Centre, Giza, Egypt; ^3^The Center for Informatics Science, Nile University, 6th of October City, Egypt; ^4^College of Biotechnology, Misr University for Science and Technology, 6th of October City, Egypt; ^5^Genetic Engineering and Biotechnology Research Institute, University of Sadat City, Sadat City, Egypt; ^6^Department of Molecular Biology, Genetic Engineering and Biotechnology Research Institute, University of Sadat City, Sadat City, Egypt

**Keywords:** cancer treatment, plant flavonoids, *Pulicaria jaubertii*, multi-kinase inhibitors, mutant K-Ras/B-Raf proteins

## Abstract

**Introduction:** Cancer is one of the most difficult challenges faced by humanity due to its many associated issues, such as inability to prevent diseases, treatment safety, and high mortality rate. In cancer, a variety of cellular signaling is activated to ensure malignancy transformation, angiogenesis and metastasis. The most efficient signaling pathway in cancer is mitogen-activated protein kinase (MAPK), which controls malignancy and regulates apoptosis.

**Methods:** Four different flavonoid glycosides have been isolated from *Pulicaria jaubertii* using the phytochemical characterization of hydro-methanol extract. The purified glycosides (PJs) were investigated for their potential repression of cancer development using human lung epithelial cells and hepatocellular carcinoma (HCC) and compared with Sorafenib (SOR), the standard systemic drug for HCC. In PJ-treated cells, the expression profile of K-Ras, B-Raf, and P53 were detected using qRT-PCR, flow cytometry, confocal microscopy and western blot. Steady-state mRNA and levels of transforming growth factor-beta (TGF-β) and interleukin 8 (IL-8) were monitored in the fluids media at different time points following treatment.

**Results:** Our results showed that the qurictine glycosides (PJ-1 and PJ-9) selectively inhibited the mutant K-Ras/B-Raf proteins expression and interaction in both cancer cells; while SOR showed obvious depletion of total Raf-1 protein in cancer cells and normal cells as well. Interestingly, the combination of PJ-1 or PJ-9 with SOR exhibited restoring cell viability of normal cells via controlling Raf-1 and P53 genes expression. Further, these identified PJ agents significantly adjusted the levels of TGF-β and IL-8 in cancer treated cells accompanied by restoring the activation of P53 expression. These findings were confirmed by docking analysis of PJs ligand and the crystal structure of K-Ras, B-Raf, and ERK transcription factor.

**Conclusion:** The current data provide novel and natural multi-kinase inhibitors with competitive regulation of the mutant proteins; K-Ras and B-Raf and sustained MAPK signaling without any detectable toxic effect in normal cells.

## Introduction

Cancer is a kind of disease that supports abnormal cell growth with the possibility of transferring to another part of the body. The different hallmarks of cancer cells include immortality, angiogenesis, and metastasis (1). Genetic alteration such as chromosomal abnormality, genetic mutation, and epigenetic modification is critical and required for cancer initiation (2). A variety of extracellular and intracellular signaling is involved in cancer diseases in which the protein kinase C (PKC) plays a crucial role in several signal transduction cascades. For instance, PKC is a family of protein kinases with a multifunctional role in the regulation of cell behaviors such as cell proliferation, differentiation, and cell death. PKC signaling includes a variety of downstream signaling transduction such as mitogen-activated protein kinase (MAPK), which includes activation of Ras oncoprotein, and PI3K/AKT/mTOR pathway (3–6). Ras protein is involved in signaling pathways that facilitate gene transcription, which is required for cell growth and differentiation. Over-expression or mutation in the Ras gene can, therefore, lead to uncontrolled cell growth and cancer development. Alteration of the Ras gene has been found in many types of cancer, including pancreatic, lung, thyroid, bladder, liver, and ovarian cancers (7–9). Noteworthy, K-Ras is a unique mutation of Ras protein that frequently occurs in human cancer and a major downstream target of epidermal growth factor (EGFR) pathway (10, 11). Activation of K-Ras stimulates B-Raf, a mutant serine-threonine protein kinase that subsequently activates genes involved in cell proliferation via Erk transcription factor. K-Ras and B-Raf mutations are the crucial mediators in EGRF signaling pathway and developmental RASopathy syndrome in human cancer (12). Protein kinase B, also known as Akt protein, is one of the proto-oncogenes that play a critical role in the induction and evaluation of cancer cells. Conversely, the Akt pathway is inactivated due to the activation of p53, the tumor suppressor gene, in normal cells (13, 14). The transforming growth factor-beta (TGF-β) is the most important signaling event that is associated with many aspects of cancer development, including malignancy, adhesion, angiogenesis, metastases, apoptosis, and immortality. Indeed, TGF-β plays dual-dependent roles in cancer; in pre-malignant cells, TGF-β regulates cell growth by its exogenous autocrine activity like tumor suppressor factors. Whereas, in a malignant tumor, TGF-β signaling promotes invasion, angiogenesis, and metastases as an oncogenic factor (15–17). Because of its pro-oncogenic function, TGF-β signaling is being considered as a prognostic biomarker for carcinogenic progress and a potential target for prevention and treatment of malignant tumor (18, 19). Importantly, the anticancer activity of plant-derived flavonoids has been reported in several studies. Recently, flavonoids exhibit a wide range of biological activities such as anti-allergic, anti-inflammatory, anti-oxidant, and anti-carcinogenetic activities. Accordingly, in the present study, we aim to highlight and confirm the role of isolated flavonoid glycosides from *Pulicaria jaubertii* as potential anti-proliferation agents using human lung epithelial cells and hepatocellular carcinoma (HCC) cell lines.

## Materials and Methods

### Cell Lines

Human lung cancer cells (A549 cells, CCL-185, ATCC) and HCC (HepG2 cells) were obtained from VACSERA (Giza, Egypt) and were grown in RPMI media (Invitrogen, Germany), which supplemented with 4 mM L-glutamine, 4 mM sodium pyruvate, 100 U/ml penicillin/streptomycin, and 2.5% of heat-treated bovine serum albumin (BSA) (Biowest, USA). Human diploid lung fibroblasts HEL-299 and normal hepatocytes cells were grown in RPMI media that contains 4 mM L-glutamine and 10% BSA. All cell lines were incubated at 37°C under 5% CO_2_ condition (20).

### Plant Materials and Fraction Methods

*Pulicaria jaubertii* was collected in 2014, from the high mountains of al Udayn, Ibb, Yemen. The whole plant air-dried powder of *P. jaubertii* (1 Kg) was extracted using hydro-methanol (70%; v/v, 6 L) at room temperature (RT) for 3 times along 3 days. The extract was concentrated under vacuum affording dry black gum extract (37.5 g). The dry extract was dissolved in a little amount of distilled water and the aqueous solution was next submitted to Polyamide 6S column chromatography and eluted with H_2_O-MeOH (1:0, 4:1, 3:2, 2:3, 1:4, 0:1 v/v) that afforded 7 major fractions (PJ-1:PJ-7). Fractions PJ-3, PJ-4, and PJ-5 were separately subjected to repeated Sephadex LH-20 column chromatography (Sigma, USA) and eluted with different mixtures of MeOH-H_2_O to afford five pure compounds (**1**, 17.2 mg), (**2**, 13.6 mg), (**3**, 11.3 mg), (**4**, 9,1 mg), and (**5**, 23.2 mg) (21).

### Chemical Treatment and Cell Viability Rate

To study the effectiveness of the purified PJ compounds and SOR treatment in cancer and normal cells, the cells were seeded in 96-well plate in a density of 10,000 cells/well and were incubated overnight at 37°C in CO_2_ incubator. Next the cells were treated with different concentrations of each PJ compound (0.1–1.5 mg/ml) and/or SOR (0.1–1 mg/ml) followed by 24 h incubation. In 6-well plates, the cells have been seeded in a density of 20 ×10^5^ cells/well followed by treatment with 100 μg/ml of each PJ composition and/or SOR. To investigate cell viability rate and cytotoxicity of chemical treatment, WST-1 assay reagent (Abcam, USA) has been used. According to manufacturer protocol, the WST-1 reagent was added to cell culture media of treated cells and incubated for 2 h followed by analyzing the amount of formazan dye by measuring absorbance at 440 nm.

### Quantitative Real Time PCR (qRT-PCR)

To quantify messenger RNA (mRNA) of indicated genes, qRT-PCR was used to perform cDNA construction and amplification in one step using the purified total RNA as a template. Total RNA from treated cells was extracted 24 h post-treatment and purified using TriZol (Invitrogen, USA) and the RNeasy Mini Kit (Qiagen, USA). The relative expression of B-Raf, P53, IL-8, and TGF-β was detected using the QuantiTect SYBR Green PCR Kit (Qiagen, USA) and oligonucleotides specific for each gene (Table 1). Level of housekeeping glyceraldehyde 3-phosphate (GAPDH) gene was used for normalization. The following mixture was prepared for each reaction; 10 μl SYBR green, 0.2 μl RNase inhibitor (20 U/μl), 0.5 μl reverse transcriptase (50 U/μl), 1 μl purified total RNA (100 ng/μl), and 1 μl from each primer up to final volume of 25 μl using RNase free water. According to the manufacturer's protocol, the following PCR parameters were used; 50°C for 30 min, 95°C for 3 min, 35 cycles (95°C for 30 s, 60°C for 15 s, 72°C for 30 s). Levels of Raf-1, P53, IL-8, and TGF-β relative to GAPDH were obtained using comparative delta-delta Ct equations (22).

**Table 1:** Oligonucleotides sequence used for detection of steady-state mRNA of the indicated genes.

**Description**	**Primer sequences 5^′^-3^′^**
Raf-sense	TTTCCTGGATCATGTTCCCCT
Raf-anti-sense	ACTTTGGTGCTACAGTGCTCA
P53-sense	GCGAGCACTGCCCAACAACA
P53-anti-sense	GGTCACCGTCTTGTTGTCCT
IL-8-sense	AAGAGAGCTCTGTCTGGACC
IL-8-anti-sense	GATATTCTCTTGGCCCTTGG
TGF-β-sense	CCGATGGGTTGTACCTTGTC
TGF-β-anti-sense	GGGCTGGGTAGAGAATGGAT
GAPDH-sense	TGGCATTGTGGAAGGGCTCA
GAPDH-anti-sense	TGGATGCAGGGATGATGTTCT

### Enzyme-Linked Immunosorbent Assay (ELISA)

ELISA test was used for quantitative measurement of TGF-β and IL-8 using human ELISA kits (Abcam 181421 and Abcam 100575, respectively). A549 cells were seeded in 96-well plat, at a density of 10 ×10^3^ cells per well and were incubated overnight. The cells were subjected to 5 μg per well (100 μg/ml) of indicated compounds and were incubated for different time points (0, 6, 12, 24, 48, and 72 h). Treated cells were lysed and loaded into the ELISA-96-well plate followed by 4 h incubation at 37°C. After washing, 100 μl of detection antibody was added to each well followed by 1-h incubation at RT. Then 200 μl of substrate solution was added to each well followed by 20 min incubation away from the light at RT. Finally, 50 μl from stop solution was added to the wells and the intensity of the color was measured at 450 nm (23–25).

### Immunofluorescent Assay

Cells were seeded in 24-well plate with sterilized coverslips at a density of 5 ×10^4^ cells per well followed by overnight incubation. Cells were overnight subjected to 100 μg/ml of the individual compound then treated cells were rinsed twice with PBS and were fixed with 4% Paraformaldehyde (PFA) for 10 min at RT. Cells were then washed with PBS, permeabilized and blocked for 10 min with PBS that contains 0.2% BSA and 0.1% Triton X-100 at RT. The cells were incubated for 1 h at RT with mouse monoclonal anti-K-Ras (Invitrogen, USA) diluted in 0.2% BSA-PBS solution followed by 1 h incubation at RT, away from the light, with goat anti-mouse IgG (Alexa Fluor 488, Abcam, USA). After washing, the cells were incubated, as described above, with rabbit monoclonal anti-B-Raf (Invitrogen, USA) followed by incubation with goat anti-rabbit IgG (Alexa Fluor 594, Abcam, USA) diluted in BSA-PBS. Samples were washed three times with PBS then incubated with the fluorescent DNA dye (DAPI) 1 μg/ml for 15 min at RT. Lastly, samples were air-dried, mounted using Mowiol mounting medium and inverted on glass microscopic slides. Cell images were captured using a laser scanning confocal fluorescence microscope with a 60X objective (Olympus Fluoview FV10i) (26). The quantification of targeted proteins was analyzed using ImageJ software. Graphs represent integrated density which reveals the mean gray value of the pixel values in a selected area related to the cell number.

### Flow Cytometry Analysis

For Raf-1, B-Raf, and phosphorylated-P53 (pho-P53) protein assay in treated cells, flow cytometry was used. Cell lines were seeded in 6-well plate at a density of 2 ×10^5^ cells per well followed by overnight incubation. Cells were treated with 100 μg/ml of each purified flavonoid and/or SOR followed by 24 h incubation at 37°C in CO_2_ incubator. To prepare treated cells for staining, the media was removed and cells were washed using PBS and trypsinized by using 100 μl trypsin. To stop trypsinization, 2 ml of complete media was added and the cells were pelleted in PBS by centrifugation and fixed by resuspension in PBS with 2% formaldehyde for 10 min. Then the cells were washed and resuspended in PBS that contains Triton-X-100 (0.1%) for permeabilization followed by centrifugation. The supernatant was removed, the pellet was resuspended in PBS that contains 1% BSA and 1:500 diluted rabbit monoclonal anti-Raf-1 (Abcam, USA) or rabbit monoclonal anti-B-Raf (Invitrogen, USA) and incubated for 1 h at RT followed by 1 h incubation, in dark, with secondary antibody (goat anti-rabbit IgG, Alexa Fluor 488). The cells then were collected and incubated for 1 h at RT with mouse monoclonal anti-pho-P53 (Cell signaling, USA). The cells were collected and washed three times using PBS. Then the cells were incubated for 1 h with goat anti-mouse IgG (Alexa Fluor 594, Abcam, USA) in dilution of 1:100. Finally, the stained cells were centrifuged and washed by PBS and were collected in 500 μl PBS for flow cytometry assay (BD Accuri 6 Plus Flow cytometry) (27).

### Western Blot

Total protein was extracted from treated cells using the RIAP lysis buffer (ThermoFisher, USA), and proteins electrophoresis were carried out using vertical Bio-Rad Mini-Protean II electrophoresis unit. Equal amounts of proteins were subjected to 15% sodium dodecyl sulfate-polyacrylamide gel electrophoresis (PAGE). Proteins were separated under reducing conditions for 3 h at 100 V and then separated proteins were transferred onto nitrocellulose membranes (Millipore, MA, USA) using Bio-Rad electroblotting system (Bio-Rad Mini Trans-Blot Electrophoretic Transfer Cell) for 3 h at 250 mA at 4°C. The membranes were blocked with 30 ml of 5% fat-skim milk in TBS containing 0.05% Tween-20 for 1 h at RT. Next, membranes were individually incubated for 1 h at RT with primary antibodies, mouse monoclonal anti-K-Ras, rabbit monoclonal anti-B-Raf, and mouse monoclonal anti-pho-P53. Finally, the membranes were washed 3 times with TBS-0.05% Tween-20 and then incubated with goat anti-rabbit secondary antibody conjugated with horseradish peroxidase. Signals were detected by the enhanced chemiluminescence system (ECL, Amersham), after soaking the membrane in the chemiluminescence solutions 1 and 2 (1:1) for minutes (26).

### Docking Analysis

The X-ray/solution structures of targeted proteins: K-Ras, B-Raf, and Erk were downloaded from the RCSB Protein Data Bank (PDB) (IDs: 5WDR, 2GX9, and 1WGR, respectively). VegaZZ program was used for removing ligands, as well as all water molecules from the crystal structures and for adding hydrogen to the proteins. The ligands and the compounds PJ-1, PJ-5, PJ-7, PJ-8, and PJ-9 were docked to the protein targets using the online docking server SwissDock. All other parameters were set as default values. Types of interactions of the docked enzyme with ligand were analyzed upon the finish of molecular docking. All graphical pictures were made using Maestro.

### Data Analysis

All data analysis, graphs, charts, and histograms were created by Microsoft Excel. All values were presented as mean ± SD and the student's two-tailed *t*-test was used for statistical analysis. A *P*-value of <0.05 was considered statistically significant. Double delta Ct analysis was used to drive the expression fold change of mRNA detected by qRT-PCR using the following equation: (1) ΔCt = Ct value for gene- Ct value for GAPDH, (2) (ΔΔCt) = ΔCt for value (experimental) –ΔCt for value (control), (3) Expression fold change = (2^−ΔΔ*ct*^) (28).

## Results

### Cytotoxic Activities of Purified Quercetin Glycosides PJs in A549 Cells

**Figure 1:**
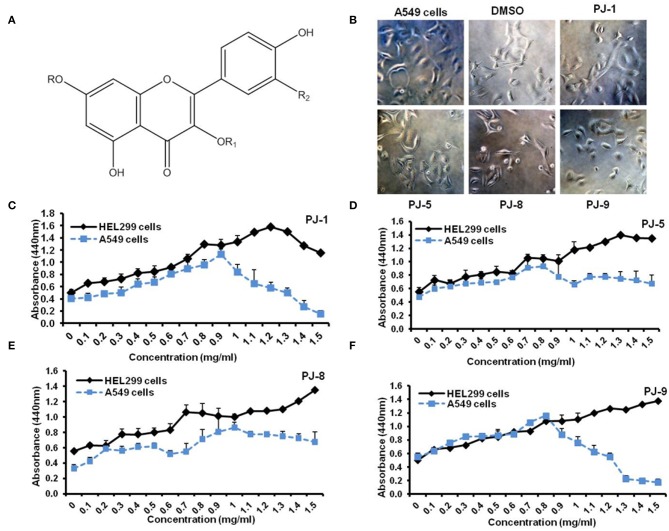
Flavonoid glycosides (PJs) structure and cell proliferation assay. **(A)** Chemical structure of isolated PJ compounds, R=H, R_1_ = β-D-glucopyranoside, R_2_ =OH — (PJ1), R=H, R_1_ = β-D-glucopyranoside, R_2_ =H — (PJ-5), R=H, R_1_ = β-D-xylopyranoside, R_2_ = OH —(PJ-8) and R= β-D-glucopyranoside, R_1_ =H, R_2_ =OH — (PJ-9). **(B)** Representative cell images reveal the cell viability of A549 cells that were pre-treated with indicated qurictine glycosides (PJ-1, PJ-5, PJ-8, and PJ-9) in comparison with DMSO-treated cells and non-treated cells (NT). **(C–F)** Calculated CC_50_-dependent cell viability rate of purified PJs on normal epithelial cells, HEL299 cells, and A549 cells that were pre-treated with different concentrations (0–1.5 mg/ml) of each PJ product using WST-1 assay.

Four known flavonoid glycosides were purified from *P. jaubertii*, namely, quercetin 3*-O*-β-D-glucopyranoside (PJ-1), kaempferol 3*-O*-β-D-glucopyranoside (PJ-5), quercetin 3*-O*-β-D-xylopyranoside (PJ-8), and quercetin 7*-O*-β-D-glucopyranoside (PJ-9) (Figure 1A). To state the potential cytotoxic effects of purified quercetin glycosides PJ-1, PJ-5, PJ-8, and PJ-9 on A549 cells and the normal lung cells HEL299, cell representative images were monitored on pre-treated cells with 100 μg/ml of each compound. The result showed that all compounds have a similar inhibitory effect on A549 cells compared with DMSO pre-treated cells (Figure 1B). The cytotoxic concentration 50% (CC_50_) of indicated quercetin glycosides was measured by WST-1 assay on A549 cells and HEL299 cells. Interestedly, the cell viability rate of treated HEL299 showed an increasing level in a dose-dependent manner; while cell viability of treated A549 cells was interrupted in the higher concentrations of both PJ-1 and PJ-9. Based on the cell viability rate, the CC_50_ of PJ-1 and PJ-9 were higher than 1,000 μg/ml (1.5 and 1.3 mg/ml, respectively), while CC_50_ of PJ-5 and PJ-8 was not detectable at higher concentration up to 1.5 mg/ml (Figures 1C–F). These findings corroborate previous findings, indicating that quercetin glycosides, PJ-1, and PJ-9 are safe at the lower and higher concentrations in normal cells and providing these reagents as efficacious anti-cancer compositions.

### Inhibition of K-Ras/B-Raf Expression and Interaction by Quercetin Glycosides; PJ-1 and PJ-9

**Figure 2:**
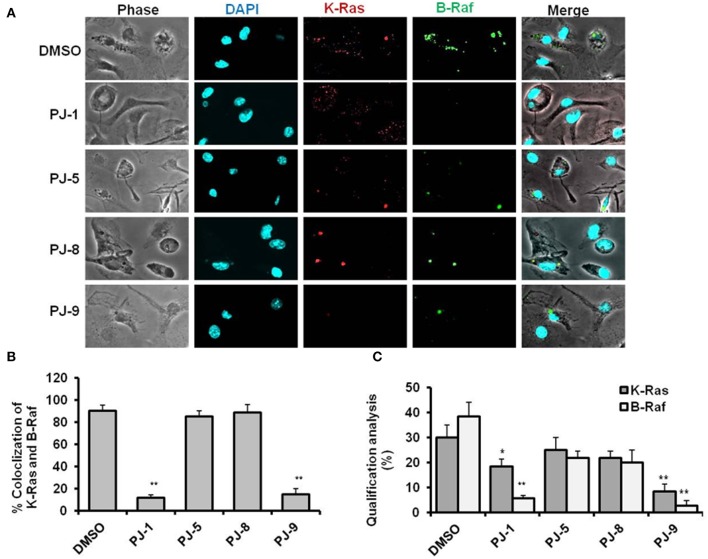
Representative confocal images of K-Ras and B-Raf proteins in PJ-treated cells. **(A)** Representative images of immunofluorescent assay indicating the levels of K-Ras (red) and B-Raf (green) in PJ-treated A549 cells compared to DMSO-treated (control). 4′,6-Diamidino-2-Phenylindole, Dihydrochloride (DAPI) was used for chromosome counterstaining (blue). **(B)** Quantification of K-Ras colocalized with B-Raf mutant proteins. **(C)** Qualification of K-Ras and B-Raf proteins intensities using ImageJ software. The error bars indicate the stranded deviation (SD) of three independent experiments. Student two-tailed *t*-test was used for significance analysis of proteins quantification values. ^*^P < 0.05 was considered statistically significant and ^**^P < 0.01 as highly significant.

To elucidate the mode of action of PJs quercetin glycosides on mutant K-Ras/B-Raf protein expression and interaction, A549 cells were cultured on coverslips in a 24-well plate at a density of 5 ×10^4^ cells per well. Then the cells were treated with 100 μg/ml of each glycoside followed by 24 h incubation. The expression of K-Ras and B-Raf corresponding proteins were monitored by using the confocal microscopy assay. The proteins intensity and colocalization of K-Ras and B-Raf were quantified using ImageJ software. Interestingly, PJ-1 (100 μg/ml) effectively suppressed the interaction between K-Ras and B-Raf indicated by the contradictory expression of both and revealed fewer colocalization event compared with DMSO treated cells (Figures 2A,B). Meanwhile, the protein quantification of both K-Ras and B-Raf was significantly decreased in PJ-1-treated cells compared with control-treated cells (Figure 2C). PJ-5 treatment revealed less inhibition of K-Ras/B-Raf colocalization and protein intensity at the same concentration in comparison with DMSO-treated cells (Figures 2A–C). Likewise, PJ-9 (100 μg/ml) efficiently reduced the expression profile and colocalization of both K-Ras and B-Raf compared to control-treated cells, whereas PJ-8 showed insufficient inhibition of indicated protein profile at the same concentration (Figures 2A–C). This result indicates that PJ-1 can inhibit the subsequent activation of B-Raf protein even with the activation of K-Ras, the upstream kinase, in A549 cells. Furthermore, PJ-9 sufficiently regulates the expression profile of both mutant K-Ras and B-Raf protein kinases and could regulate the MAPK/ERK signaling pathway in A549 cells.

### PJ-1 and PJ-9 Regulate B-Raf and Restore P53 Expression Profile in Cancer Cells

**Figure 3:**
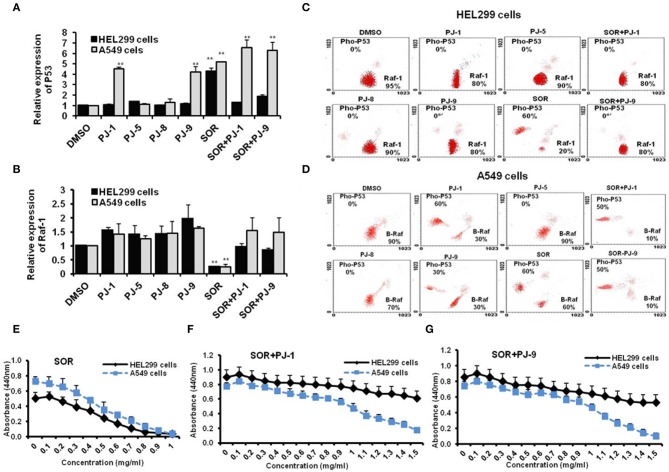
Expression profile of Raf-1, mutant B-Raf, and phosphorylated P53 in SOR and/or PJ-treated cells. **(A)** Steady-state mRNA of P53 gene indicated by fold change in SOR and/or PJ-treated cells compared with DMSO-treated cells. **(B)** Steady-state mRNA of Raf-1 gene indicated by fold change in SOR and/or PJ-treated cells in comparison with DMSO-treated cells. Levels of GAPDH-mRNA were used as an internal control. Error bars indicate the SD of three independent experiments. Student two-tailed *t*-test was used for significance analysis of cycle threshold (Ct) values. **(C)** Steady flow cytometry fractionation quantifies kinetic profile of Raf-1 and pho-P53 proteins in treated HEL299 cells in comparison with DMSO treated cells. **(D)** Quantified protein profile of both B-Raf and pho-P53 in treated A549 indicated by flow cytometry and compared to DMSO-treated cells. **(E–G)** Cell viability rate of normal and cancer cells that were pre-treated with SOR, SOR+PJ-1, and SOR+PJ-9, respectively. Error bars indicate the SD of four different replicates. ^**^P < 0.01 was considered as highly significant.

Steady-state mRNA of P53 and Raf-1 in SOR and PJs-treated cells was quantified by using q-RT-PCR. Interestingly, the steady-state mRNA of P53 was significantly increased (up to 7-fold) in cancer cells that were pre-treated with PJ-1 and PJ-9 or SOR in comparison with control-treated cells. In normal cell, P53 expression profile was significantly increased upon SOR treatment, while its relative expression was reduced in cells that were pre-treated with SOR+PJ-1 or SOR+PJ-9. Meanwhile, the other quercetins, PJ-5 and PJ-8 showed a negligible effect on P53 expression profile compared with DMSO-treated cells (Figure 3A). Importantly, the relative expression of the Raf-1 gene was constant in all PJ-treated cells, while its expression was decreased in SOR-treated cells and restored in cells pre-treated with SOR+PJ-1 or SOR+PJ-9 (Figure 3B). Furthermore, flow cytometry was used for quantification of total Raf-1, phosphorylated P53 and mutant B-Raf proteins at a single-cell level via a combination of phospho-specific antibodies to stain treated cells. HEL299 and A549 cells were individually treated with indicated quercetin glycosides and DMSO for 24 h to establish the kinetic profile of total Raf-1, pho-P53, and B-Raf expression. To quantify the level of pho-P53 stabilization in treated cells, the percentage of positive events was correlated to the relative fluorescent intensity of the Alexa Fluor signal. The expression profile of pho-P53 and Raf-1 proteins were assessed in normal cells as a response to SOR and PJs treatment. As expected, SOR treatment strongly inhibited the expression profile of Raf-1 protein in treated cells accompanied by increasing levels of pho-P53 proteins. In contrast, PJs treatment showed increasing levels of Raf-1 protein profile but not pho-P53 protein. The combination of SOR and PJ-1 or PJ-9 successfully regulates activation of P53 protein and restores the kinetic expression of Raf-1 (Figure 3C). Furthermore, the kinetic expression of both pho-P53 and B-Raf were detected in SOR and PJ-treated cancer cells. Interestingly, the kinetic expression of pho-P53 protein was increased up to 60, 60, and 30% in cells that were pre-treated with SOR, PJ-1, and PJ-9, respectively. Meanwhile, the kinetic expression of B-Raf protein was reduced to almost 30% in the same treated cells, whereas other qurictine glycosides, PJ-5 and PJ-8 had an obscure effect on the kinetic expression of both pho-P53 and B-Raf compared with control-treated cells. The combination of SOR with PJ-1 or PJ-9 strongly inhibited the kinetic expression of the mutant protein B-Raf that accompanied by increasing levels of pho-P53 protein expression (Figure 3D). To check the cytotoxic effect of SOR in cancer and normal cells, the CC_50_ of SOR was considered by WST-1 assay which revealed decreasing levels of cell viability rate in a dose-dependent manner and indicated that the CC_50_ of SOR is equal 500 μg/ml in cancer and normal cells (Figure 3E). In contrast, the cell viability rate of normal cells was regular when treated with SOR+PJ-1 or SOR+PJ9, while cell viability of cancer cells was disturbed at the highest concentration of either SOR+PJ-1 or SOR+PJ-9 (1.2 mg/ml) (Figures 3F,G). Together, flow cytometry and cell viability experiments further confirm the ability of both PJ-1 and PJ-9 to restore P53 activity and to regulate kinetic expression of the mutant protein, B-Raf in cancer cells with a dynamic effect in normal cells.

### PJ-1 and PJ-9 Modulate Cell Viability and Adjust IL-8 Dependent TGF-β Production in Cancer Cells

**Figure 4:**
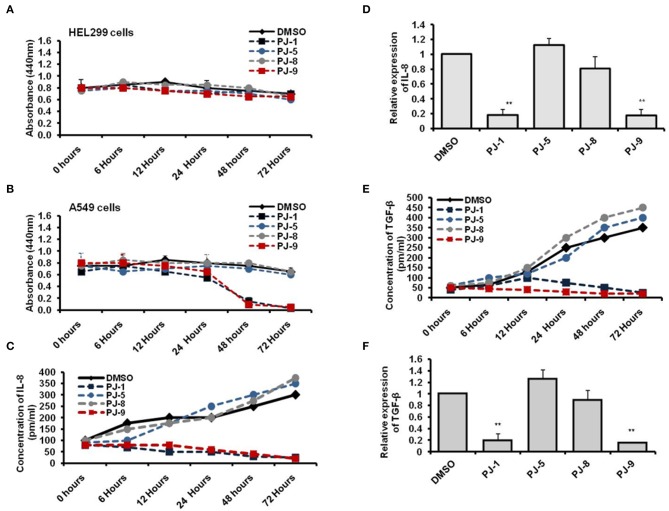
Levels of produced IL-8 and TGF-β in PJ-treated A549 cells. **(A)** Cell viability rate of the normal cells that were subjected to 100 μg/ml of each PJ compound at the indicated time points. **(B)** Cell viability rate of the cancer cells that were subjected to the same concentration of each PJ compound at the indicated time points. Error bars indicate the SD of two independent experiments. **(C)** The concentration of produced IL-8 (pm/ml) in the fluids media of A549 cells that were subjected to 100 μg/ml of each PJ glycoside at the indicated time points compared with DMSO-treated cells**. (D)** Fold change in steady-state mRNA of IL-8 in PJ-treated cells related to DMSO-treated cells and indicated by qRT-PCR. **(E)** Level of produced TGF-β (pm/ml) from A549 cells that were subjected to 100 μg/ml of each PJ glycoside for the indicated time points compared with DMSO-treated cells. **(F)** Fold change in steady-state mRNA of TGF-β in PJ-treated cells related to DMSO-treated cells and indicated by qRT-PCR. GAPDH-mRNA was used as an internal control for the qRT-PCR test. Error bars reveal the SD of three independent experiments. Student two-tailed *t*-test was used for significance analysis of Ct values indicated by qRT-PCR. ^**^P < 0.01 was considered as highly significant.

To assess the time-dependent efficiency of PJ glycosides on treated cells, normal and cancer cells were subjected to 100 μg/ml of each purified PJ compound and incubated for different time points including 6, 12, 24, 48, and 72 h. Cell viability of treated was monitored by WST-1 at the indicated time points. The result showed that all indicated PJs have no cytotoxic effect on the normal cells, HEL299 when compared with DMSO-treated cells, while PJ-1 and PJ-9 showed an obvious effect on cancer cell at 48 h following treatment (Figures 4A,B). To achieve the potential influence of PJ compositions on the regulation of pro-inflammatory cytokines secretion, levels of produced IL-8 and TGF-β were monitored in supernatant fluids of treated cells. Accordingly, A549 cells were subjected to 100 μg/ml of each PJ glycoside in a time course treatment for 6, 12, 24, 48, and 72 h at which the concentration of both IL-8 and TGF-β were assessed using the ELISA test. Further, the relative steady-state mRNA of both IL-8 and TGF-β was quantified in 24 h upon treatment. The result showed that PJ-1 and PJ-9 at 100 μg/ml dramatically down-regulated the release of IL-8 in a timed dependent. Moreover, the relative steady-state mRNA of IL-8 was significantly decreased in cells that were subjected to the same PJs compared to control-treated cells (Figures 4C,D). Additionally, levels of produced TGF-β was depleted in cells that were subjected to PJ-1 or PJ-9 in a timed course experiment as well as the relatively steady-state of its mRNA upon overnight treatment (Figures 4E,F). Together these findings indicate that PJ-1 and PJ-9 can modulate cell viability of cancer cell and can adjust the production of IL-8 and TGF-β in a time-dependent manner.

### PJ-1 and PJ-9 Regulate K-Ras/B-Raf Mitogen-Activated Proteins in HepG2 Cells

**Figure 5:**
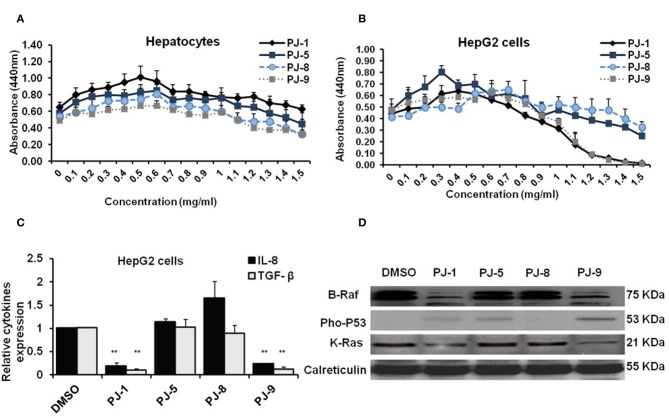
Biological activities of PJ compositions in normal hepatocytes and HepG2 cells. **(A)** Cells viability rate and indicated CC_50_ of purified PJ glycosides on normal hepatocytes cells that were pre-treated with different concentrations (0–1.5 mg/ml) of each PJ. **(B)** Cell viability rate and CC_50_ of PJ products on treated-HepG2 cells using WST-1 assay. Error bars indicate the SD of four different replicates. **(C)** Fold change in steady-state mRNA of IL-8 and TGF-β in PJ-treated cells related to DMSO-treated cells and indicated by qRT-PCR. GAPDH-mRNA was used as an internal control for the qRT-PCR test. Error bars reveal the SD of three independent experiments. Student two-tailed *t*-test was used for significance analysis of Ct values indicated by qRT-PCR. **(D)** Immunoblotting assay indicates mutant K-Ras, B-Raf, and phospho-P53 proteins in PJ-treated cells compared to DMSO-treated cells. The calreticulin was served as an internal control. ^*^P < 0.05 was considered statistically significant and ^**^P < 0.01 as highly significant.

To figure out whether PJ glycosides commonly have such a regulatory effect on cancer cell proliferation, HepG2 cells and normal hepatocytes cells were used. First, normal hepatocytes and HepG2 cells were subjected to different concentrations of PJ compositions (0–1.5 mg/ml) to detect the CC_50_ of each compound. The result showed that the cytotoxic concentration of all indicated PJs is not detectable in normal hepatocytes or may >1.5 mg/ml; while on cancer cells, only PJ-1 and PJ-9 showed the cytotoxic effect at the higher concentrations including 1.3 and 1.5 mg/ml, respectively (Figures 5A,B). Second, the relative steady-state mRNA of IL-8 and TGF-β was significantly down-regulated in cancer cells that were overnight subjected to PJ-1 or PJ-9 in comparison with control-treated cells (Figure 5C). Importantly, by immunoblotting assay, the expression of mutant K-Ras corresponding protein and its down-stream target B-Raf have been decreased in cells that were subjected to PJ-1 or PJ-9, while the expression of pho-P53 protein has been restored in cells that were subjected to PJ-1 or PJ-9 in comparison with control-treated cells (Figure 5D). These results further confirm the previous findings which indicated that PJ-1 and PJ-9 can regulate the pro-inflammatory cytokine secretion via depletion of K-Ras/B-Raf expression and rescue of P53 activity in cancer cells.

### Binding Based-Cross Docking Analysis of PJ Ligands and MAPK/ERK Chain Proteins

**Figure 6:**
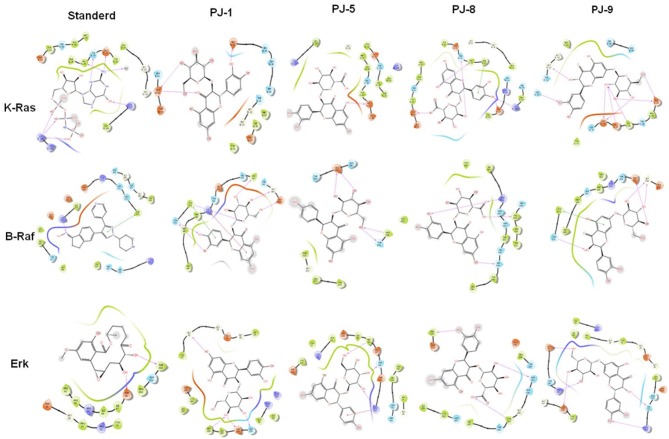
Binding discrimination of PJ ligands with MAPK/ERK proteins: The possible hydrophobic interaction and binding affinity between quercetin glycosides PJ-1, PJ-5, PJ-8, and PJ-9 and the crystal structure of K-Ras, B-Raf, and Erk chain protein indicated by pharmacophore mapping snapshot.

At last, to further confirm our findings, the possible binding affinity and docking interaction of PJ ligands and mutant MAPK proteins have been investigated. At the minimum binding energy, K-Ras protein was successfully docked with quercetin glycosides, PJ-1 and PJ-9. The possible binding affinity of PJ-1 at K-Ras active sites showed four hydrogen bonds and hydrophobic interaction with protein residue PHE 28 (Figure 6). As shown in Figure 6, K-Ras protein residues ASP 33, ILE 36, GLU 37, ASP 38, GLN 61, and GLY 12 formed a hydrogen bond with a PJ-9 ligand molecule. Meanwhile, PJ-9 showed relative binding discrimination in comparison with Phosphoaminophoshphonic Acid-Guanylate Ester (GNP), the official standard, which showed minimum binding energy of −8.0 kcal/mol. As a co-factor of K-Ras protein, B-Raf showed good binding affinity with PJ-1 and PJ-9 ligands at the minimum energy compared to binding affinity with official standard Canonical SMILES. A total of six hydrogen bonds and two hydrophobic interactions were found in docked PJ-1 with Raf-1 protein structure including (GLU 545, GLY 544, LEU 433, TYR 574, ASN 576, LYS 439, and LYS 439). PJ-9 showed four hydrogen bonds and hydrophobic interaction with B-Raf, including the residues (GLY 544, GLU 437, TYR 574, and ASN 576). Erk crystal structure showed good binning discrimination with PJ-1 and PJ-9 ligands compared to the binding affinity with its standard ligand HYM. A total of four hydrogen bonds were indicated between PJ-1 ligand and Erk protein residues GLY 35, GLN 103, ASP 104, and MET 106. PJ-5 formed three hydrogen bonds and hydrophobic interaction with Erk residues MET 106, THR 108, and LYS 112, whereas a total of five hydrogen bonds were formed between PJ-9 ligand and Erk residues ARG 65, GLU 69, LYS 149, SER 151, and ASP 165 (Figure 6). Together the current docking analysis indicates that quercetin glycoside PJ-9 has multiple inhibitory effects on K-Ras, B-Raf-1, and Erk protein kinases, while PJ-1 has a competitive regulatory effect on B-Raf and Erk protein, respectively.

## Discussion

In this study, we sought to highlight the role of naturally purified qurictine glycosides in cancer treatment as safe and efficacious agents. Therefore, the biological activity of purified flavonoid glycosides form *P. jaubertii* (PJs) was deeply investigated in different cancer cells, including A549 and HepG2 cells in comparison with normal lung cells, HEL299, and normal hepatocytes. The potential cytotoxic concentration of each isolated glycoside on treated cells was higher than 1,000 μg/ml. Only PJ-1 and PJ-5 showed a negative influence on cancer cells at the concentration of 1.3 and 1.5 mg/ml, respectively. Thus, we investigated the pharmacological activities of individual glycosides at a lower concentration (100 μg/ml). Our findings showed a competitive inhibitory effect on both mutant K-Ras and B-Raf proteins interaction by PJ-1 and PJ-9 agents in both indicated cancer cells. Moreover, PJ-1 and PJ-9 treatment reduced the production level of pro-inflammatory cytokine IL-8 in the regulation of TGF-β accompanied by an accumulation of P53 gene expression. These data indicate that quercetin glycosides PJ-1 and PJ-9 that are isolated and purified from *P. jaubertii* can regulate cancer cell division via activating programmed cell death and preventing the resulting inflammation. Furthermore, the repression events of IL-8 and TGF-β in treated cells exhibit the potential regulatory influence of PJ-1 and PJ-9 in the angiogenesis and metastases of cancer cells. Typically, canonical activation of MAPK signaling begins at the cell surface and subsequently stimulates a variety of protein kinases and ultimately modulates several transcription factors. The MAPK pathway is required to control many cellular processes, including cell differentiation, cell division, cell proliferation, programmed cell death, and inflammation. MAPK/ERK signaling pathway includes activation of Raf protein kinase, MEK1/2, and the transcription factor ERK1/2 (29, 30). This pathway is activated in cancer cells due to the activation of mutant K-Ras and mutant B-Raf proteins which responsible for the limitation of programmed cell death and for raising the production level of TGF-β and IL-8 (31, 32). TGF-β is a multi-functional transforming growth factor controlling the differentiation of stem cells, chemotaxis and metastasis in cancer cells (33). IL-8 is a chemokine that plays a key role in the angiogenesis events in cancer. TGF-β, tumor necrosis factor (TNF-α) and IL-1β can induce IL-8 production from different cells, including endothelial cells, macrophages, and epithelial cells (34, 35). IL-8 is highly expressed in different cancer cells such as breast, ovarian, lung, and liver cancers. Interestingly, our findings indicate that the purified PJ agents (PJ1 and PJ9) can disturb sustained MAPK signaling which plays a major role in the mortality of cancer cells, via targeting of both mutant K-Ras and mutant B-Raf proteins. Further, PJ1 and PJ9 successfully regulate the production level of pro-inflammatory cytokines (IL-8 and TGF-β), which are responsible for the angiogenesis and metastases of cancer cells. Nevertheless, targeting of IL-8 and TGF-β parallel with the regulation of mutant K-Ras/B-Raf mitogen proteins by PJs flavonoid glycosides could provide a better strategy for developing secure anti-cancer agents. It is well-known that carcinogenicity in humans has multiple steps initiated with genetics alternation and followed by malignancy transformation, angiogenesis, and metastases. Lacking specific gene alternation and epigenetic profiling associated with malignancy makes it difficult to characterize an effective and specific treatment for gene abnormalities in cancer. For instance, several DNA methyltransferase inhibitors, including 5-aza-2′-deoxycytidine, sufficiently prevented the carcinogenicity; however, they also led to hypomethylation and the expression of other silent genes in normal cases (36, 37). For controlling malignancy transformation and cell proliferation, several inhibitors that are targeting the Raf kinase family have been identified such as sorafenib. Noteworthy, Raf protein kinases are the essential factors of the MAPK pathway that regulates cell division (38). Stimulation of Raf kinase proteins is required activation of the upstream Ras protein, which in turn activates the other downstream factors MEK1/2 and ERK1/2. Nevertheless, Raf inhibitors lack long-term therapeutic efficiency and raise the elusive resistance during treatment (39). A variety of angiogenesis inhibitors have been approved, including Axitinib, Lenvatinib, and Cabozantinib, based on their efficiency in the regulation of vascular endothelial growth factor (VEGF) and its receptors (40–42). Notably, critical side effects are associated with VEGF inhibitor treatment including renal dysfunction, hypertension atherosclerosis and the brain syndrome, reversible posterior leukoencephalopathy (43–45). Therefore, the search for safe and effective treatment of cancer cells is an important requirement to face the disease. Natural occurring products could be the most effective solution for controlling cancer cells with minimal possible side effects. In summary, in the current work, we provide novel multi-kinase inhibitors that are isolated from *P. jaubertii* with regulatory effects on sustained MAPK signaling and pro-inflammatory cytokines secretion in cancer cells. These glycoside agents regulate the mutant K-Ras/B-Raf proteins and rescue the expression of tumor suppressor P53 gene in treated cells accompanied by a low production level of IL-8 and its regulatory factor TGF-β. Such a mechanism indicated that PJ agents are secure for the treatment of cancer cells with an obvious effect on cell proliferation, inflammatory events, and metastases.

## Conclusion

Our findings showed a competitive inhibitory effect on mutant K-Ras/B-Raf proteins expression and interaction by PJ-1 and PJ-9 agents that are isolated from *P. jaubertii* in both in A549 and HepG2 cells. Moreover, PJ-1 and PJ-9 treatment reduced the production level of pro-inflammatory cytokine IL-8 in the regulation of TGF-β accompanied by an accumulation of P53 gene expression. These data indicate that quercetin glycosides PJ-1 and PJ-9 can regulate cancer cell division by repairing the sustained Ras/Raf/Erk signaling pathway and regulate programmed cell death. Furthermore, the repression of IL-8 and TGF-β signaling events in treated cells exhibits the potential regulatory influence of indicated PJ-1 and PJ-9 in the angiogenesis and metastases of cancer cells. Such a mechanism indicated that PJ agents are secure in the treatment of cancer cells with an obvious effect on cell proliferation, inflammatory events, and metastases.

## Data Availability Statement

The raw data supporting the conclusions of this manuscript will be made available by the authors, without undue reservation, to any qualified researcher.

## Author Contributions

HK and AA conceived and designed the research. HK, AA, RT, AG, EA, KE, OK, SM, and AS performed the experiments. HK prepared and wrote the manuscript. All authors commented on the manuscript and have given their final approval for submission.

### Conflict of Interest

The authors declare that the research was conducted in the absence of any commercial or financial relationships that could be construed as a potential conflict of interest.
